# PReS13-SPK-1033: Neonatal lupus

**DOI:** 10.1186/1546-0096-11-S2-I5

**Published:** 2013-12-05

**Authors:** R Cimaz

**Affiliations:** 1 Pediatrics, University of Florence, Florence, Italy

## 

Children born from mothers positive for autoantibodies against SSA/Ro and/or anti-SSB/La ribonucleoproteins may develop heart conduction tissue damage resulting in atrioventricular block and/or transient skin rash, liver enzyme abnormalities and anaemia/thrombocytopenia. Additional transient electrocardiographic abnormalities (sinus bradycardia, QT interval prolongation) have been reported. Such clinical and laboratory manifestations are included in the so-called Neonatal Lupus Syndromes, independently whether the mother is suffering from a systemic autoimmune disease or is totally asymptomatic.

The prevalence of the congenital heart block is around 2%, of neonatal lupus rash around 20%, while laboratory abnormalities in asymptomatic babies can be detected in up to 30% of cases. The risk of recurrence of complete heart block is almost ten times higher in the following pregnancies. Most of the mothers are asymptomatic at delivery and are identified only by the birth of an affected child. Their longterm outcome is generally more reassuring than previously assumed and arthralgias and xerophtalmia are the commonest symptoms.

A standard therapy for heart blocks detected *in utero *is still matter of investigation, although fluorinated corticosteroids have been reported to be effective on myocarditic signs when present. Serial echocardiograms and obstetric sonograms, performed at least every two weeks starting from the 16 weeks' gestation, are recommended in anti-Ro/SSA positive pregnant women: the goal is to detect early fetal abnormalities, that might precede complete atrioventricular block and that might be a target of preventive therapy.

Transplacental passage of maternal anti-SSA/SSB IgG is thought to be pivotal in inducing tissue damage. However, the discordant appearance of the syndrome in twins does suggest a role also for foetal or environmental factors.

## Disclosure of interest

None declared.

